# C1q, a small molecule with high impact on brain development: putative role for aging processes and the occurrence of Alzheimer’s disease

**DOI:** 10.1007/s00406-021-01273-9

**Published:** 2021-05-13

**Authors:** Christian Rupprecht, Rainer Rupprecht, Gerhard Rammes

**Affiliations:** 1 Experimental Neuropharmacology, Department of Anesthesiology, Technical University Munich, Ismaninger Strasse 22, 81675 Munich, Germany; 2 Department of Psychiatry and Psychotherapy, University of Regensburg, Regensburg, Germany

Numerous proteins have been suggested to play a role in neurodegenerative disorders such as Alzheimer’s disease (AD). A well-known protein, the complement protein C1q, has meanwhile entered the field and might stimulate further research in this area.

C1q is a small molecule in the shape of a bunch of flowers [1] and is widely known for its role within the native immune system. As initiator of the antibody-dependent pathway it recognizes pathogenic structures in the blood stream, which in turn promotes their tagging and ultimately phagocytosis. For that reason C1q is also known as an “eat me” signal. Nowadays, emerging studies indicate that C1q may play a similar role within the brain. This in turn includes on the one hand a physiological function during brain development and, on the other hand, under pathological conditions, e.g., acute brain damage or AD, C1q may even promote neurodegeneration.

## Complement dependent synaptic pruning

Neuronal plasticity is a major feature of brain development and brain function. It is determined by both biological and environmental factors such as the everyday use of brain functions.

The breakdown of synapses in the context of autoimmune processes, e.g., during brain development, is also known as synaptic pruning. Two complement proteins are involved in the microglia-dependent pruning mechanisms. C3b, the active form of the C3 protein, can opsonize neurons and is recognized by microglia by means of its C3 receptor. Furthermore, the C1q protein affects synaptic pruning in two ways: on the one hand, synapses tagged by C1q are recognized by the C1q receptor expressed on microglia. On the other hand, C1q may increase the amount of activated C3 [2, 3]. Whereas, during brain development this process allows the reduction of the formation of excessive dendritic spines and, consequently, the elimination of immature synapses and brain circuits [3], during adulthood synaptic pruning is far less pronounced under normal conditions [4]. However, it is of note that the expression of brain C1q shows a rather different pattern across the life span.

## C1q increases exponentially during normal aging

Both Stephan et al. [5] and Reichwald et al. [6] reported an increase of C1q expression during the life span in C57BL/6 mice. In both studies, C1q levels raised slowly in young but rather sharply increased in older animals, suggesting an exponential age-dependent time course of C1q expression.

**Fig. 1:**
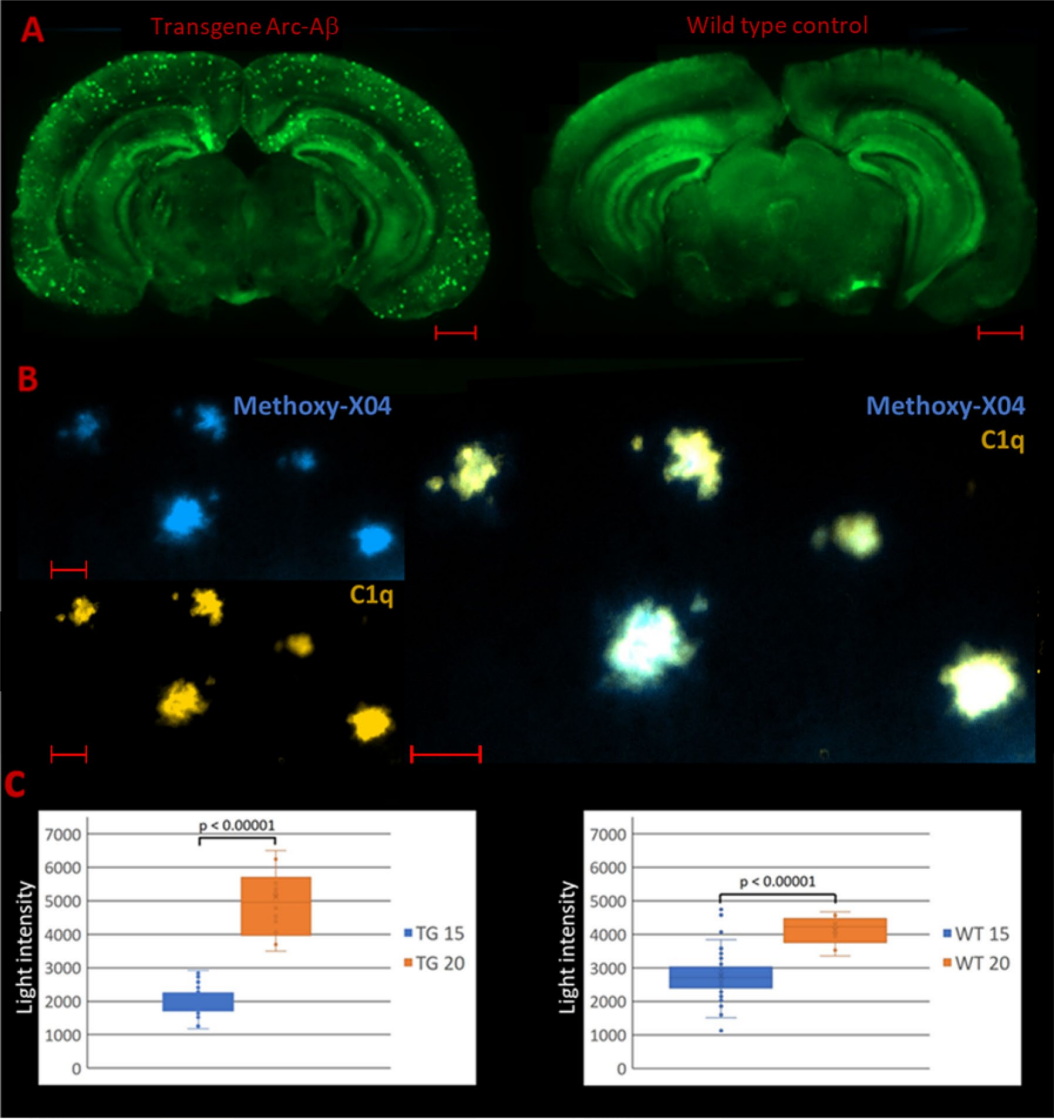
C1q staining in transgenic C57BL/6 Arc-Aβ mice and corresponding wild type control animals. **a** Immunofluorescence images of PFA fixed free-floating stained brain sections using the monoclonal anti-C1q antibody Abcam 182541: C1q staining (*green*) is distributed over the entire brain. C1q plaques only occur in the transgenic Alzheimer’s disease mouse model Arc-Aβ (scale bars: 1000 µm). **b** Colocalization of C1q and β-amyloid plaques: C1q depositions (*orange*) continuously overlap with Methoxy-X04 stained β-amyloid plaque accumulations (scale bars: 50 µm). **c** Light intensity measurements of C1q stained brain sections: C1q expression within the dentate gyrus differs significantly between 15- and 20-months-old animals (Mann–Whitney *U* test: *p* < 0.00001). This effect is even more pronounced in the transgenic mouse model (*TG* transgenic, *WT* wild type)

Moreover, our own studies in the same mouse strain indicated an increase of about 50% within the dentate gyrus during a time interval of only 5 months in older wild type animals (Fig. 1c). Using an immunofluorescence method, even regional differences in age-dependent C1q deposition became apparent. Figure 1a reveals the overall distribution of C1q over the brain. By means of this C1q staining method various brain areas can be visualized. As such, a clear distinction is feasible between regions with a high C1q expression such as the dentate gyrus and some parts of the hippocampus, e.g., cornu ammonis (CA) layers, and areas with low expression, e.g., the thalamus region. Our data revealed that those areas with already pronounced C1q expression such as the hippocampus and especially the dentate gyrus are largely susceptible for an age-dependent C1q expression (Fig. 1c). Since C1q is involved into the loss of synapses and in view of this prominent effect of age particularly in the hippocampus, the question arises whether C1q may be also involved in the pathophysiology of neurodegenerative disorders such as Alzheimer's disease (AD).

## Putative role of C1q for Alzheimer’s disease

Alzheimer’s disease pathology may affect brain C1q levels in two ways. On the one hand, the effect of age may even be enhanced during this disease. It has been reported that APP23 mice at the age of 9 months showed significant greater C1q mRNA levels compared to the wild type control, which is even more pronounced with increasing age [6]. This is in line with our own light intensity measurement data (Fig. 1c). Transgenic Arc-Aβ mice, which are characterized by overexpressing human APP695 with Swedish (K670N/M671L) and Arctic (E693G) mutations and constitute a valid model for pathological beta amyloid deposition [7], showed a markedly greater increase of C1q during aging in comparison to wild type control animals.

On the other hand, the Alzheimer pathology itself may influence C1q expression. For example, in mice treated intraventricularly with β-amyloid (Aβ) a significant increase of C1q levels occurred [8].

Reichwald et al. [6] as well as our own data strongly argue for an age dependent C1q expression pattern. At younger age, transgenic mouse models for Alzheimer’s disease are characterized by rather low C1q levels. However, in aged animals this expression pattern is completely different and is reflected by rather elevated brain C1q levels. Further investigations are needed to resolve this age-dependent shift in expression patterns in AD models.

Besides the effect on soluble C1q AD also promotes the accumulation of C1q in form of aggregates. C1q plaques only occur in animal models of AD and are mainly located in the cortex and the hippocampus (Fig. 1a). Interestingly, those C1q aggregates appear to overlap completely with Aβ plaques unraveled in respective colocalization experiments (Fig. 1b). Moreover, also in human postmortem brain tissue of patients suffering from AD it has been shown that increased C1q expression is positively correlated with Aβ plaques [9].

## C1q is involved in the pathophysiology of Alzheimer’s disease

In addition to the descriptive data reporting a correlation between C1q levels, age and AD discussed above, there is also evidence for a direct involvement of C1q in the pathophysiology of this disease. Hong et al. [8] demonstrated that early loss of synapses during AD is dependent on the complement system. After contact with oligomeric Aβ C1q expression did not only increase quantitatively but also showed an increased binding to synaptic material. As a result, oligomeric Aβ lead to a reduction in dendritic spine density, which could not be observed in the absence of C1q.

## Conclusion and outlook

In summary, it can be assumed that synaptic pruning mechanisms can be aberrantly reactivated particularly during older age and thereby contribute to neurodegeneration. This is in line with the well-known phenomenon that age constitutes a major risk factor for neurodegenerative disorders such as AD. Complement proteins such as C1q may play a role as “eat me” signals in this context and opsonize synapses for microglia-mediated phagocytosis. These mechanisms constitute a good example of the cooperation of the cellular and non-cellular immune responses in the context of inflammatory processes. Future research should address the following questions: What is the exact role of C1q during age and neurodegeneration in the human brain? Is an increase of C1q triggering neurodegeneration or is it simply a consequence of other ongoing neurodegenerative processes? May C1q serve as a general marker for neurodegeneration or is there a difference between various forms of dementia? Can C1q serve as a putative biomarker either by quantification in CSF alone or in conjunction with other complement markers [10] or is it accessible in molecular neuroimaging studies, e.g., by positron emission tomography (PET)? May C1q even constitute a putative therapeutic target? In conclusion, C1q and the complement system will add new avenues to the already complicated puzzle underlying aging and neurodegenerative disorders such as Alzheimer’s disease.
